# CALIBER: a phase II randomized feasibility trial of chemoablation with mitomycin‐C vs surgical management in low‐risk non‐muscle‐invasive bladder cancer

**DOI:** 10.1111/bju.15038

**Published:** 2020-04-03

**Authors:** A. Hugh Mostafid, Nuria Porta, Joanne Cresswell, Thomas R.L. Griffiths, John D. Kelly, Steven R. Penegar, Kim Davenport, John S. McGrath, Nicholas Campain, Peter Cooke, Shikohe Masood, Margaret A. Knowles, Andrew Feber, Allen Knight, James W.F. Catto, Rebecca Lewis, Emma Hall

**Affiliations:** ^1^ Royal Surrey County Hospital NHS Foundation Trust Guildford UK; ^2^ Institute of Cancer Research London UK; ^3^ South Tees Hospitals NHS Foundation Trust Middlesbrough UK; ^4^ University Hospitals of Leicester NHS Trust Leicester UK; ^5^ University College London London UK; ^6^ Gloucestershire Hospitals NHS Foundation Trust Cheltenham UK; ^7^ Royal Devon and Exeter NHS Foundation Trust Exeter UK; ^8^ Royal Wolverhampton Hospitals NHS Trust Wolverhampton UK; ^9^ Medway NHS Foundation Trust Gillingham UK; ^10^ University of Leeds Leeds UK; ^11^ Action Bladder Cancer UK Gloucestershire UK; ^12^ University of Sheffield Sheffield UK

**Keywords:** non‐muscle‐invasive bladder cancer, chemoablation, surgery, mitomycin‐C, randomized trial, #BladderCancer, #blcsm

## Abstract

**Objectives:**

To evaluate the activity of intravesical mitomycin‐C (MMC) to ablate recurrent low‐risk non‐muscle‐invasive bladder cancer (NMIBC) and assess whether it may enable patients to avoid surgical intervention for treatment of recurrence.

**Patients and Methods:**

CALIBER is a phase II feasibility study. Participants were randomized (2:1) to treatment with four once‐weekly MMC 40‐mg intravesical instillations (chemoablation arm) or to surgical management. The surgical group was included to assess the feasibility of randomization. The primary endpoint was complete response to intravesical MMC in the chemoablation arm at 3 months, reported with exact 95% confidence intervals (CIs). Secondary endpoints included time to subsequent recurrence, summarized by Kaplan–Meier methods.

**Results:**

Between February 2015 and August 2017, 82 patients with visual diagnosis of recurrent low‐risk NMIBC were enrolled from 24 UK hospitals (chemoablation, *n = *54; surgical management, *n *=28). The median follow‐up was 24 months. Complete response at 3 months was 37.0% (20/54; 95% CI 24.3–51.3) with chemoablation and 80.8% (21/26; 95% CI 60.6–93.4) with surgical management. Amongst patients with complete response at 3 months, a similar proportion was recurrence‐free by 12 months in both groups (84%). Amongst those with residual disease at 3 months, the 12‐month recurrence‐free proportion was lower in the surgical management group (40.0%) than in the chemoablation group (84%). Recruitment stopped early as chemoablation did not meet the prespecified threshold of 45% complete responses at 3 months.

**Conclusion:**

Intravesical chemoablation in low‐risk NMIBC is feasible and safe, but did not demonstrate sufficient response in the present trial. After chemoablation there may be a reduction in recurrence rate, even in non‐responders, that is greater than with surgery alone. Further research is required to investigate the role and optimal schedule of neoadjuvant intravesical chemotherapy prior to surgery for NMIBC.

## Introduction

Bladder cancer is the ninth most common cancer worldwide [1], and most frequently presents as non‐muscle‐invasive bladder cancer (NMIBC). Approximately 50% of patients with bladder cancer have low‐risk NMIBC [2], with a 0.8–6% risk of progression to muscle‐invasive disease or bladder cancer death within 5 years and a relatively high rate of local recurrence, 46–62% [2, 3, 4]. Half of recurrences occur within the first year of follow‐up [5]. The discomfort and inconvenience of managing NMIBC recurrence, combined with cost, are the key issues for patients and healthcare providers managing low‐risk NMIBC [6, 7, 8].

Guidelines recommend annual cystoscopy for 5 years for low‐risk NMIBC [2]. Treatments for local recurrence include transurethral resection and cystodiathermy under general anaesthesia, laser ablation under local anaesthesia and watchful waiting [9, 10]. This variety reflects the indolent nature of low‐risk NMIBC and lack of high‐quality evidence about the optimal management.

Several small studies have demonstrated promising results for intravesical chemotherapy alone (chemoablation) as an alternative to surgical management for NMIBC. The optimal schedule and its effectiveness in achieving a complete response in low‐risk NMIBC are unclear. Reviews of chemoablation (including >1200 patients with varying risk and different chemotherapy regimens) suggest the complete response rate is ~50%, with the therapeutic effect sustained for at least 2 years [11, 12]. These data suggest chemoablation may be a viable treatment for low‐risk NMIBC.

To inform trial design, 100 patients undergoing surveillance for low‐risk NMIBC were surveyed. They had concerns regarding inpatient surgical management of recurrence under general anaesthesia and stated a preference for a non‐surgical outpatient option. A focus group of patients with NMIBC was then held to discuss potential trial designs, at which, based on available data [11, 12], chemoablation was confirmed as an attractive alternative to surgical management for recurrent low‐risk NMIBC, and suitable success criteria for a phase II trial were agreed.

CALIBER was therefore developed to investigate intravesical chemoablation as an alternative to surgical management for recurrent low‐risk NMIBC, incorporating patient‐reported outcomes to assess the acceptability to participants of the treatments.

## Patients and Methods

### Trial Design, Management and Governance

CALIBER (NCT02070120) is a phase II multicentre feasibility study. A two‐stage randomized design was used to establish the chemoablation response rate whilst obtaining prospective surgical management data and assessing the feasibility of randomization to treatments for any subsequent comparative trial. Recruitment was planned to continue seamlessly between stages 1 and 2.

The trial was approved by the Medicines and Healthcare Products Regulatory Authority and South Central – Hampshire‐B Research Ethics Committee (ref: 14/SC/1223, approved 29 August 2014), sponsored by The Institute of Cancer Research (ICR) and conducted according to the principles of good clinical practice. The Clinical Trials and Statistics Unit at the ICR (ICR‐CTSU) co‐ordinated the study and data collection, and conducted statistical analysis. The trial management group was overseen by independent data monitoring and trial steering committees.

### Patients

Eligible patients had previously diagnosed, histologically confirmed, low‐risk NMIBC with visual diagnosis of recurrence. Patients were aged >16 years, with an European Organisation for Research and Treatment of Cancer (EORTC) risk of recurrence score ≤6 [2] (this criterion was revised in December 2016 from ≤5, as a result of inadvertent exclusion of patients for whom chemoablation may be an appropriate treatment option), with no history of high grade/≥T1 or non‐urothelial bladder cancer. Patients with prior treatment of the recurrence or contraindication to trial treatment were excluded. All participants provided written informed consent.

### Treatment Allocation and Study Procedures

Patients were recruited at UK NHS hospitals and allocated by the ICR‐CTSU to either chemoablation or surgical management in a 2:1 ratio. Treatment allocation was by minimization with a random element, with balancing factors of treating site and recurrence history (first or further recurrence). Treatment allocation was not blinded.

Patients in the chemoablation group received four once‐weekly intravesical instillations of 40 mg mitomycin‐C (MMC) as outpatients, in accordance with local policy. No dose reductions were permitted. Patients assigned to surgical management underwent the local standard technique for treatment of recurrence; a single instillation of 40 mg MMC within 24 h postoperatively was permitted.

A cystoscopy was conducted 3 months after treatment completion to assess response visually and to biopsy the tumour bed. Subsequent cystoscopic follow‐up was at 6 (if disease detecded at 3 months) and 12 months after treatment, and annually thereafter.

### Outcomes

The primary endpoint was complete response to chemoablation at 3 months post‐treatment, defined as an absence of any bladder tumour both by visual assessment and biopsy.

Secondary endpoints included time from end of treatment to subsequent recurrence, subsequent transurethral resection of bladder tumour (TURBT)/biopsy rates after the 3‐month disease assessment, safety, and patient‐reported health‐related quality of life (HRQoL) outcomes.

Adverse events were assessed at end of treatment and at 3 months, using National Cancer Institute Common Terminology Criteria for Adverse Events (CTCAE) version 4.0. HRQoL was assessed with the EORTC’s general quality‐of‐life questionnaire, the QLQ‐C30 [13], and the NMIBC‐specific module (QLQ‐NMIBC24) [14]. The primary objective of the HRQoL study was to assess differences between groups in the global quality‐of‐life scale of the QLQ‐C30. Questionnaires were completed by patients at baseline, 3, 6 and 12 months.

### Statistical Considerations

CALIBER was designed to exclude a complete response rate of <45% in the chemoablation group. Using a Simon two‐stage optimal design [15], complete response in at least 26/51 chemoablation patients was required in stage 1. Prior to stage 1 analysis, the design was adapted to reduce stage 2 sample size and remove the randomization (Data S1). In the revised design, with 85% power and an α value = 0.10, complete response in at least 31/60 chemoablation patients was required at the end of stage 2. The total target recruitment was 89 patients, 63 patients in the chemoablation group (accounting for 5% non‐compliance) and 26 in the surgical management group (stage 1 control group).

Efficacy outcomes were analysed on the evaluable population, i.e. patients with 3‐month assessment data who received their allocated treatment. Sensitivity analyses on the per‐protocol and eligible populations were performed (Table S1). Safety analyses were conducted according to treatment received.

Complete response rate was calculated based on (i) no disease on visual assessment at 3‐month cystoscopy, and (ii) where 3‐month biopsy was performed, no disease on histopathology assessment. Patients with visually detected disease, or positive histology when visually clear, were classified as not responding. Both definitions were considered for the stage 1 stop/go decision. Complete response rates were presented with exact binomial 95% CIs. The trial was not powered for the direct comparison of complete response rate between treatment groups and no formal statistical comparisons of the primary endpoint were planned.

Time to first subsequent recurrence after response status assessment at 3 months was summarized using Kaplan–Meier methods, and treatment groups were compared by the stratified log‐rank test, adjusting by response status at 3 months. The four groups defined by the combination of treatment and response status at 3 months were compared using the log‐rank test. Frequency of subsequent NMIBC recurrence/TURBT was summarized by treatment; worst CTCAE grade adverse event was summarized by timepoint and treatment received. Treatment comparisons used chi‐squared or Fisher's tests as appropriate. Statistical comparisons for the secondary endpoints were considered exploratory.

Standard algorithms were used to derive scores from and handle missing HRQoL data [16]. Change from baseline was calculated and summarized descriptively at each subsequent timepoint, with means and 99% CIs. A larger confidence level was chosen for HRQoL endpoints to account for multiplicity across subscales and timepoints.

Analyses were based on a data snapshot taken on 10 October 2018, triggered once all patients had at least 12 months of follow‐up (or earlier if lost to follow‐up), and performed using STATA version 15.0 (StataCorp LLC, College Station, TX) [17].

## Results

### Participants

**Fig. 1:**
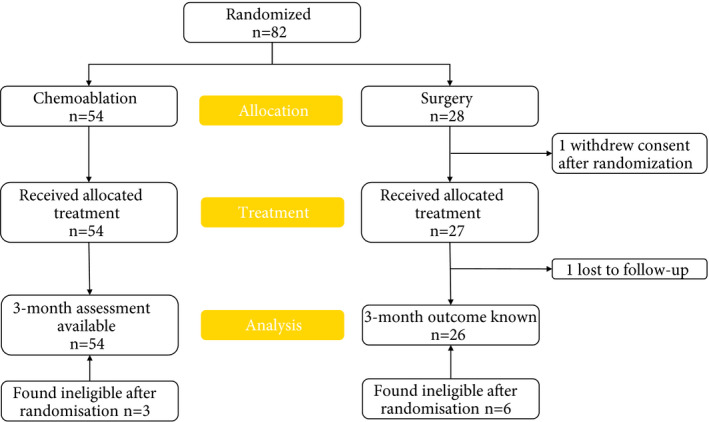
CONSORT diagram. Eighty patients were included in the primary and efficacy endpoints’ analysis: two patients without a 3‐month assessment in the surgical management group were excluded (one withdrew from trial treatment after randomization, one was lost to follow‐up before 3 months). All patients for whom there were completed post‐treatment and/or 3‐month adverse event forms were included in the safety analyses (*N* = 81). Nine patients (three surgical management, six chemoablation) were found ineligible after randomization but were included in all analyses in accordance with the CALIBER Statistical Analysis Plan.

Eighty‐two patients were enrolled in the study (chemoablation, *n = *54; surgical management, *n =*28) from 24 UK sites between February 2015 and August 2017 (Fig. 1). Fifty‐six percent (82/145) of eligible patients reported on sites’ screening logs consented to participation. CALIBER ceased recruitment in August 2017, after the Independent Data Monitoring Committee concluded the trial should stop for futility based on stage 1 complete response rates.

Baseline features were evenly matched across treatment groups (Table 1). In the chemoablation group, 53 patients (98%) received all four planned instillations, with one patient receiving three. In the surgical management group, 27 patients received surgery, of whom 16 (57%) received diathermy (Table 2).

**Table 1:** Baseline characteristics of CALIBER participants.

	Surgical management group (*N* = 28)	Chemoablation group (*N* = 54)	All patients (*N* = 82)
**Gender, *n *(%)**			
Male	23 (82.1)	40 (74.1)	63 (76.8)
Female	5 (17.9)	14 (25.9)	19 (23.2)
**Age, years**			
Mean (sd)	69.3 (11.5)	73.4 (7.6)	72.0 (9.2)
Median (Q1–Q3)	70.7 (61.1–77.1)	72.5 (68.8–78.3)	72.4 (66.8–77.9)
**Number of tumours at trial entry, *n *(%)**			
1	21 (75.0)	47 (87.0)	68 (82.9)
2–7	7 (25.0)	7 (13.0)	14 (17.1)
**Maximum tumour diameter at trial entry, *n *(%)**			
<3 cm	27 (96.4)	54 (100.0)	81 (98.8)
≥3 cm	1 (3.6)	0 (0.0)	1 (1.2)
**Recurrence rate at trial entry, *n *(%)**			
≤1 year	27 (96.4)	49 (90.7)	76 (92.7)
>1 year*	1 (3.6)	5 (9.3)	6 (7.3)
**Number of previous occurrences of NMIBC** ^†^ **, *n *(%)**			
1	15 (53.6)	30 (55.6)	45 (54.9)
2	8 (28.6)	12 (22.2)	20 (24.4)
3	4 (14.3)	4 (7.4)	8 (9.8)
4	0 (0.0)	3 (5.6)	3 (3.7)
≥5	1 (3.6)	5 (9.3)	6(7.3)
**Prior MMC (single instillation) , *n *(%)**			
Yes	19 (67.9)	33 (61.1)	52 (63.4)
No	8 (28.6)	18 (33.3)	26 (31.7)
Unknown	1 (3.6)	3 (5.6)	4 (4.9)
**Grade at original diagnosis, *n *(%)**			
G1	15 (53.6)	22 (40.7)	37 (45.1)
G2	13 (46.4)	32 (59.3)	45 (54.9)
**Risk score at trial entry, *n *(%)**			
2	10 (35.7)	21 (38.9)	31 (37.8)
3	10 (35.7)	24 (44.4)	34 (41.5)
5	5 (17.9)	3 (5.6)	8 (9.8)
6	2 (7.1)	3 (5.6)	5 (6.1)
8*	1 (3.6)	3 (5.6)	4 (4.9)

MMC, mitomycin C; NMIBC, non‐muscle‐invasive bladder cancer; Q1, first quartile, 25% percentile; Q3, 3rd quartile, 75% percentile.

* Patients found ineligible after randomization, due to incorrect calculation of the risk score at site. ^†^Including diagnosis; overall (since diagnosis).

**Table 2:** Surgical management group: details of surgical technique and histology at trial entry.

	Surgical management group (*N *= 28)	
**Type of surgery, *n*(%)**		
Diathermy	16(57.1)	
TURBT	12(42.9)	
**Single postoperative MMC instillation given, *n*(%)**		
Yes	3(10.7)	
Stage*, *n*(%)		
Benign	3(10.7)	
Ta	18(64.3)	
Grade*, *n*(%)		
Benign	3(10.7)	
G1	6(21.4)	
G2	11(39.3)	
GX	1(3.6)	

MMC, mitomycin‐C; TURBT, transurethral resection of bladder tumour. * Only 21 with histological confirmation.

### Response Rates

The stage 1 stop/go decision was based on the first 51 evaluable chemoablation patients: 18 complete responses were reported by visual and histopathology assessment (where available) with 23 complete responses reported by visual assessment alone. The criterion to proceed to stage 2 was not met by either definition of complete response.

Complete response rate in the chemoablation group overall was 37% (20/54; 95% CI 24–51) by visual and histopathology assessment and 48% (26/54; 95% CI 34–62) by visual assessment alone. Complete response rate was 81% (21/26; 95% CI 61–93) in the surgical management group by visual and histopathology assessment.

**Fig. 2:**
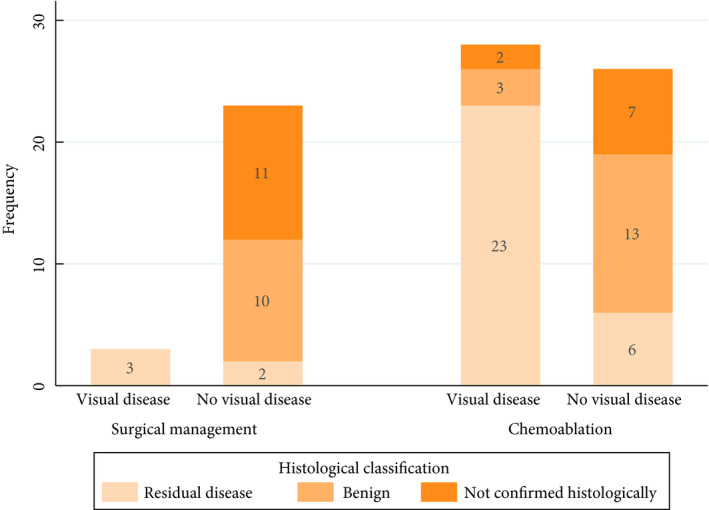
Response at 3‐month assessment: visual vs histological confirmation.

Figure 2 shows the concordance between visual and histopathology assessment. In the chemoablation group, 28/54 patients (52%) had visible disease at 3 months (no complete response), with 23/28 confirmed histologically. Of 26/54 patients (48%) with no visible disease, 6/26 had disease confirmed on biopsy. In the surgical management group, 3/26 patients (12%) had visible disease at 3 months, all confirmed histologically; 2/23 patients with no visible tumour had residual disease confirmed on biopsy. Three‐month histology was unavailable for nine patients in the chemoablation group and 11 in the surgical management group. Table 3 summarizes disease found at 3 months.

**Table 3:** Three‐month assessment: details of surgical technique and histology.

	Surgical management group		Chemoablation group		All patients	
	*n*(%)		*n*(%)		*n*(%)	
Patients with disease present at 3 months (visual and histologically, where available)	5(100)		34(100)		39(100)	
**Treatment for residual disease**						
Diathermy	1(20)		11(32.4)		12(30.8)	
TURBT	3(60)		19(55.9)		22(56.4)	
Biopsy alone	1(20)		3(8.8)		4(10.3)	
Cystoscopy alone	0(0)		1(2.9)		1(2.6)	
**Single postoperative MMC instillation given**						
Yes	0(0)		2(5.9)		2(5.1)	
**Number of tumours**						
1	5(100)		20(58.8)		25(64.1)	
2–7	0(0)		12(35.3)		12(30.8)	
Unknown	0(0)		2(5.9)		2(5.1)	
**Maximum tumour diameter**						
<3 cm	4(80)		29(85.3)		33(84.6)	
≥3 cm	1(20)		2(5.9)		3(7.7)	
Unknown	0(0)		3(8.8)		3(7.7)	
**Stage**						
Benign	0(0)		3(8.8)		3(7.7)	
Ta	5(100)		27(79.4)		32(82.1)	
Ta + CIS	0(0)		1(2.9)		1(2.6)	
CIS	0(0)		1(2.9)		1(2.6)	
Unknown	0(0)		2(5.9)		2(5.1)	
**Grade**						
Benign	0(0)		3(8.8)		3(7.7)	
G1	0(0)		10(29.4)		10(25.6)	
G2	4(80)		13(38.2)		17(43.6)	
G3	1(20)		3(8.8)		4(10.3)	
GX	0(0)		1(2.9)		1(2.6)	
Unknown	0(0)		4(11.8)		4(10.3)	
**Disease location**						
Same as trial entry	5(100)		32(94.1)		37(94.9)	
Different location	0(0)		2(5.9)		2(5.1)	

CIS, carcinoma *in situ*; MMC, mitomycin‐C, TURBT, transurethral resection of bladder tumour.

### Recurrences Subsequent to the 3‐Month Disease Assessment

With a median (interquartile range) follow‐up at time of data snapshot of 24 (15–29), months, 27 patients had NMIBC recurrences after their 3‐month disease assessment. In the chemoablation group, 16 patients (30%) had at least one NMIBC recurrence, with two (4%) experiencing more than one. Eleven surgical management patients (39%) had at least one subsequent NMIBC recurrence, with four (14%) experiencing more than one. Five chemoablation patients (9%) and six surgical management patients (21%) underwent TURBT. No statistically significant differences were found between the groups.

**Fig. 3:**
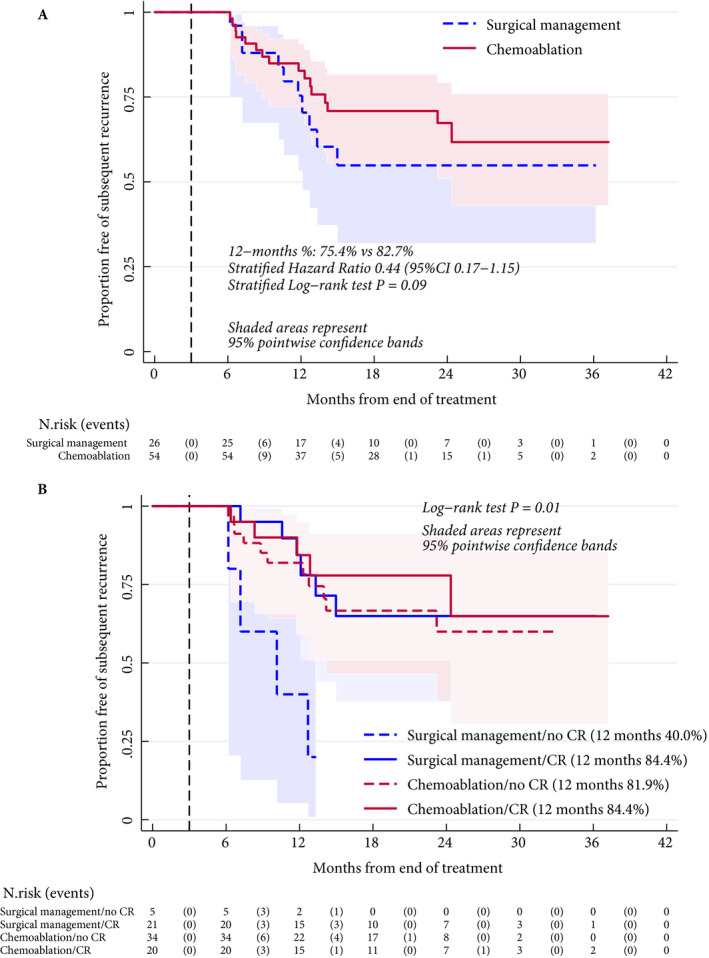
Kaplan–Meier estimate of proportion of patients free of subsequent recurrence after 3‐month disease assessment, by allocated treatment (**A**) and by allocated treatment and disease status (**B**). Patients who had a second primary cancer or died for reasons other than bladder cancer without a prior recurrence were censored at date of second primary or date of death. Stratified log‐rank test and stratified Cox model to explore the differences between treatment groups were used as appropriate to account for disease response status at 3 months (**A**). When treatment and disease status were combined to form four groups, these were compared by log‐rank test (not stratified). Proportional hazards were tested using Schoenfeld residuals.

One patient had a second primary cancer diagnosed before their NMIBC recurrence and was censored in the analysis of time to first post‐3 month recurrence. No significant difference was observed between treatment groups in recurrence rates over time (Fig. 3A). When explored by disease status and treatment at 3 months (Fig. 3B), surgical management patients with disease at 3 months did significantly worse, while the proportion free of subsequent recurrence at 12 months was similar in patients who underwent surgery and were disease‐free at 3‐months and in the chemoablation group, with or without disease at 3 months (*P* = 0.01).

### Progression Rate and Overall Survival

No patient experienced disease stage progression, although five patients had grade progression to carcinoma *in situ* and/or G3Ta at 3 months (Table 3). Two patients (one in each group) died during follow‐up, both from cardiac events not considered disease‐related; both had complete response at 3 months.

### Safety and Tolerability

Post‐treatment adverse event data were available for 81 patients. No serious adverse events or grade 3–4 adverse events were reported. Grade 2 adverse events were reported for 14/81 patients (17%), and for 29/81 patients (36%) a worst grade of 1 was reported. No differences between groups were found (Tables S2–S4). In the surgical management group, 7/28 patients (25%) experienced complications prior to discharge from surgery, mostly haematuria (six patients; 21%).

### Health‐Related Quality of Life

**Fig. 4:**
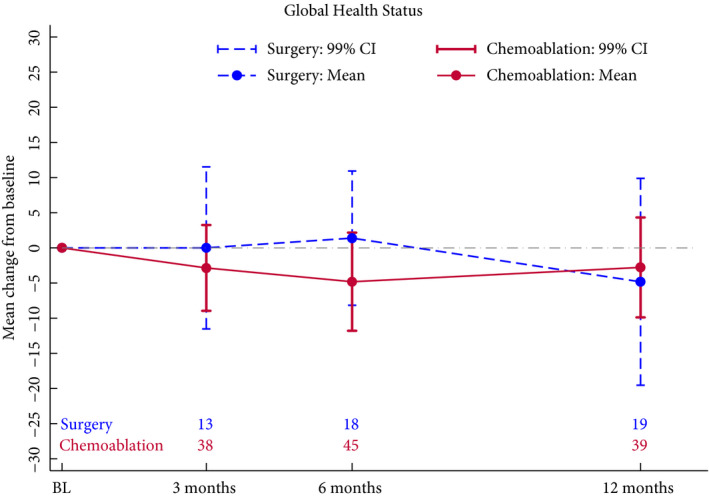
Health‐related quality of life: change from baseline in QLQ‐C30 global health scale. High score at any timepoint represents high quality of life. Positive change from baseline (calculated score at timepoint – score at baseline) represents improvement in quality of life. Questionnaire return rates were 91% at baseline, 72% at 3 months after end of treatment, 92% at 6 months, and 85% at 12 months. BL, baseline.

Seventy‐eight patients consented to participate in the optional HRQoL sub‐study (51 in the chemoablation, 27 in the surgical management group). The two treatment groups exhibited similar HRQoL throughout follow‐up, both in global quality of life and other key subscales of interest (Figs 4 and S1, S2).

## Discussion

We demonstrated the feasibility of randomization between surgical and medical management of low‐risk NMIBC. Chemoablation with four MMC instillations was well tolerated. The predefined criterion for progression to stage 2 was not met and the trial closed early, but a sustained reduction in recurrence rate was suggested. HRQoL was not substantially impacted by either treatment.

To our knowledge, this is the first study to measure the effect of chemoablation using histological rather than visual criteria. Complete response rates in both groups were lower than expected when compared to previous studies reporting visual complete response only [11, 12]. Based on our findings, visual complete response should be used with caution as a primary endpoint in NMIBC trials, although its pragmatic use in a clinical setting is probably acceptable.

At 12 months, recurrence rate was similar between patients with complete response at 3 months in both groups (16%). Rates were also similar in patients who ‘failed’ chemoablation and were ‘salvaged’ by surgical management at 3 months. By contrast, patients who ‘failed’ surgical management without prior intravesical chemoablation had a 12‐month recurrence rate of 60%, although caution is needed because of the small size of the groups.

The use of four instillations of MMC was chosen pragmatically to fit into the UK national 31‐day target for cancer surgery and avoid delaying surgery if there was no response. A more intensive or extended regimen may result in improved response rates and any further research should consider this. Our results suggest that four MMC instillations may have some chemo‐protective effect against low‐risk NMIBC recurrence. There remains a group of frail patients who tolerate surgery poorly, for whom a near 50% chance of complete ablation of visible tumours may be beneficial in terms of safety and improved quality of life.

A particular challenge for trials in low‐risk NMIBC is that the diagnosis can only be confirmed after tissue examination from TURBT; therefore, we could only recruit patients with a previous low‐risk NMIBC diagnosis who had experienced recurrence. To ensure consistency in definition of low‐risk NMIBC across multiple hospitals we used the EORTC risk score tables [18] rather than the NMIBC guideline risk categories of the European Association of Urology (EAU) [2]. These constraints had important consequences; although 50% of newly diagnosed patients with NMIBC are low risk, over two‐thirds never have any subsequent recurrence [2], whilst those that do (those eligible for this study) are re‐classified as intermediate‐risk patients, both according to EAU guidelines and the EORTC risk tables. The results should therefore be interpreted in this context.

The trial has a number of weaknesses. It was not powered for direct comparison of response rate between randomized groups, limiting ability to definitively identify differences between treatments. The study population probably reflects a group of patients with intermediate‐, rather than low‐risk NMIBC, limiting ability to extrapolate results to newly diagnosed low‐risk NMIBC. To assess potential comparators for phase III, the control arm permitted different surgical options, including biopsy with diathermy, potentially underestimating the benefits of an expertly conducted TURBT. Only three patients in the surgical group (11%) received a postoperative MMC instillation; had all patients in the surgical management group received this, the observed complete response to surgery may have been higher and the subsequent recurrence rate reduced. Finally, there was relatively poor compliance with the biopsy at 3 months, so visual assessment of response was not verified by histology for every patient.

Alternative strategies for managing low‐risk NMIBC include active surveillance [9] and office fulguration. Whilst active surveillance appears safe, our patient focus group indicated this was not a popular strategy. Office fulguration is popular in some countries because it avoids general anaesthesia and is therefore cost‐effective, but it is not popular amongst patients in the UK and is often painful, particularly for elderly patients. Moreover, in the surgical arm of CALIBER, 57% of patients had fulguration (rather than TURBT) and nearly 20% had residual disease at 3 months, which calls into question the effectiveness of using this strategy alone.

Ultimately, all three strategies have an important role to play in reducing the burden of treatment on frail patients undergoing low‐risk NMIBC surveillance. One could consider chemoablation in frail patients presenting with multifocal or very large papillary tumours prior to TURBT in the expectation that some will have their tumour burden reduced at surgery. Our results indicate that a neoadjuvant course of intravesical chemotherapy, given over a short period, is well tolerated and may provide additional therapeutic benefit over surgical management alone.

In conclusion, low‐risk NMIBC management with chemoablation as an alternative to TURBT is feasible and safe, but our study did not reach the prespecified level of complete response. Nevertheless, after chemoablation there appears to be a sustained reduction in recurrence rate that is greater than with surgical management alone. Further research is required to investigate the role and optimal schedule of neoadjuvant therapy prior to TURBT.

## Conflict of Interest

Mr. Mostafid reports grants from NIHR, during the conduct of the study; personal fees from Astrazeneca, personal fees from Olympus, personal fees from Cepheid, personal fees from Medac, outside the submitted work. Prof Hall reports grants from Department of Health, during the conduct of the study; grants and non‐financial support from Merck Sharp & Dohme, grants and non‐financial support from Astra Zeneca, grants from Janssen‐Cilag, grants and non‐financial support from Bayer, grants from Kyowa Hakko UK, grants from Alliance Pharma (previously Cambridge Laboratories), grants from Aventis Pharma Limited (Sanofi), grants from Cancer Research UK, grants from Accuray Inc., outside the submitted work. Prof. Catto reports personal fees from Astra Zeneca, personal fees from Janssen, personal fees from Roche, personal fees from Ferring and personal fees from MSD outside the submitted work. Prof. Knowles reports grants from Cancer Research UK, outside the submitted work.

**Abbreviations**
MMC: mitomycin‐C
NMIBC: non‐muscle invasive bladder cancer
ICR: Institute of Cancer Research
ICR‐CTSU: Clinical Trials and Statistics Unit at the ICR
EORTC: European Organization for Research and Treatment of Cancer
TURBT: transurethral resection of bladder tumour
HRQoL: health‐related quality of life
CTCAE: Common Terminology Criteria for Adverse Events
EAU: European Association of Urology
NIHR: National Institute for Health Research

## Supporting information


**Data S1.** Revised CALIBER design following challenges in recruitment.
**Table S1.** Complete response rates three months after end of treatment – sensitivity analyses.
**Table S2.** Worst CTCAE grade adverse event reported by visit.
**Table S3.** Worst CTCAE grade treatment emergent adverse events.
**Table S4.** Worst CTCAE grade treatment emergent adverse events by type of event.
**Fig. S1.** HRQoL Change from baseline in QLQ‐C30 Physical function.
**Fig. S2.** HRQoL Change from baseline in QLQ‐NMIBC24 Urinary symptoms.
