# Supplementary material for: CALIBER: a phase II randomized feasibility trial of chemoablation with mitomycin‐C vs surgical management in low‐risk non‐muscle‐invasive bladder cancer
Source: BJU Int. 2020 Apr 3;125(6):817–26. doi: 10.1111/bju.15038 (PMC7318672; doi:10.1111/bju.15038)
Supplement: Supplementary file 1 — Data S1. Revised CALIBER design following challenges in recruitment. Table S1. Complete response rates three months after end of treatment – sensitivity analyses. Table S2. Worst CTCAE grade adverse event reported by visit. Table S3. Worst CTCAE grade treatment emergent adverse events. Table S4. Worst CTCAE grade treatment emergent adverse events by type of event. Fig. S1. HRQoL Change from baseline in QLQ‐C30 Physical function. Fig. S2. HRQoL Change from baseline in QLQ‐NMIBC24 Urinary symptoms. [file BJU-125-817-s001.docx]

## Supplementary material: Revised CALIBER design following challenges in recruitment

*Original sample size (from protocol v1.0 06/08/2014)*

Following consultation with patient representatives, CALIBER was designed to exclude a CR rate of less than 45%. This was on the basis that if the CR rate is less than 45%, chemoablation would not be an attractive alternative to surgical management as it would delay rather than prevent surgical intervention in the majority of patients and hence would be unlikely to reduce the burden of treatment of recurrence. If the CR rate is>60% then the strategy of chemoablation would warrant further investigation. Using a Simon’s 2 stage phase II optimal design (to allow early stopping for futility) with α=0.05, 90% power, p0=0.45, p1=0.60 the required sample size is 51 chemoablation patients in the first stage. If fewer than 26 CRs are seen in chemoablation patients in the first stage, then recruitment would cease (there having been no previous break in recruitment, to allow determination of CR in stage 1 patients, on completion of accrual to stage 1). At the end of the second stage, if at least 58/110 chemoablation patients have a CR then it would be concluded that chemoablation demonstrated adequate activity to warrant further investigation. An allocation ratio of 2:1 to the chemoablation group was selected to maximise information in the experimental group whilst providing contemporaneously collected information in an unbiased control group to enable informal comparisons that would support development of a phase III trial. Therefore, inflating to account for 5% noncompliance, and to include a control group, this gives a total target recruitment of 174 patients, 116 in the chemoablation group (54 in stage 1; 62 in stage 2) and 58 in the surgical management group (27 in stage 1; 32 in stage 2).

*Revised sample size (implemented in protocol v6.0 20/06/2017)*

Due to slower than anticipated accrual and with advice and approval of the Independent Data Monitoring and Trial Steering Committees, the 2 stage trial design was adapted. This adaption was made without knowledge of the CR rate in stage 1 i.e. prior to the decision to stop/go at the completion of stage 1. Based on good acceptance rates amongst eligible patients (approximately 56% as at February 2017 as reported on screening logs), the TSC recommended that the control group could be dropped for stage 2 – the feasibility of randomisation being proven during stage 1. To address recruitment timelines, the TSC advised that the overall power and significance levels could be relaxed, whilst maintaining the original stage 1 decision rule, to achieve a reduced overall total sample size.

Following completion of recruitment to stage 1 (51 evaluable chemoablation patients) and in the absence of any safety concerns raised by the Independent Data Monitoring Committee, recruitment to stage 2 would commence with all patients receiving chemoablation. With 51 chemoablation patients recruited in stage 1 and an additional 9 chemoablation patients recruited in stage 2, the adapted 2-stage design retains p0=0.45 and p1=0.60 and the threshold for activity at stage 1 (stop/go criteria) of at least 26 responders in 51 chemoablation patients and provides 85% power and 10% one-sided significance. If at the end of stage 2, at least 31/60 chemoablation patients had a CR then it would be concluded that chemoablation demonstrated adequate activity to warrant further investigation.

Therefore, nine additional chemoablation patients would be required in stage 2 (giving a total of 60 chemoablation patients) with an overall target sample size of 89 patients, including the control group patients at stage 1 (26 patients) and allowing a 5% drop out (unevaluable) rate in the chemoablation group.

## Supplementary Table 1. Complete response rates three months after end of treatment – sensitivity analyses

Sensitivity analyses of the primary endpoint have been performed on the per protocol and the eligible populations. In addition, the following sensitivity analyses have been performed:

- Sensitivity analysis 1, surgery group: excluding evaluable patients found to be benign at baseline
- Sensitivity analysis 1, chemoablation group: evaluable patients with visual disease at three months found to be benign are considered CR in the combined visual/histological assessment.
- Sensitivity analysis 2, surgery group: Exclude from analysis two evaluable patients who received 6-course MMC following surgery and before the 3-month check.

|  | **Surgery** | | | | **Chemoablation** | | | |
| --- | --- | --- | --- | --- | --- | --- | --- | --- |
|  | **N** | **CR** | **Rate** | **95% CI** | **N** | **CR** | **Rate** | **95% CI** |
| **Per protocol population** | *Exclude ineligible, 3-m visit deviations,  benign at baseline* | | | | *Exclude ineligible, 3-m visit deviations* | | | |
| Visual assessment only | 19 | 18 | 94.7% | 74.0-99.9 | 43 | 20 | 46.5% | 31.1-62.3 |
| Visual and histological assessment (where available) | 19 | 17 | 89.5% | 66.9-98.7 | 43 | 15 | 34.9% | 21.0-50.9 |
| **Eligible population** | *Exclude ineligible patients* | | | | *Exclude ineligible patients* | | | |
| Visual assessment only | 23 | 21 | 91.3% | 72.0-98.9 | 48 | 23 | 47.9% | 33.3-62.8 |
| Visual and histological assessment (where available) | 23 | 19 | 82.6% | 61.2-95.0 | 48 | 18 | 37.5% | 24.0-52.6 |
| **Sensitivity analysis 1** | *Exclude patients benign at surgery* | | | | *Classify benign residual disease as  CR in combined assessment* | | | |
| Visual assessment only | 24 | 21 | 87.5% | 67.6-97.3 | 54 | 26 | 48.1% | 34.3-62.2 |
| Visual and histological assessment (where available) | 24 | 19 | 79.2% | 57.8-92.9 | 54 | 23 | 42.6% | 29.2-56.8 |
| **Sensitivity analysis 2** | *Exclude patients who received  MMC before 3 months* | | | |  |  |  |  |
| Visual assessment only | 24 | 21 | 87.5% | 67.6-97.3 |  |  |  |  |
| Visual and histological assessment (where available) | 24 | 19 | 79.2% | 57.8-92.9 |  |  |  |  |

CR: Complete Response; CI: confidence interval

**Supplementary Table 2 - Worst CTCAE grade adverse event reported by visit**

|  |  | **Surgery** | | **Chemoablation** | | **Total** | | **p-value** |
| --- | --- | --- | --- | --- | --- | --- | --- | --- |
|  | **CTCAE grade** | **N** | **%** | **N** | **%** | **N** | **%** |  |
| **Pre-randomisation** | **Total** | **28** | **100.0%** | **54** | **100.0%** | **82** | **100.0%** | 0.63 |
|  | 0 | 21 | 75.0% | 42 | 77.8% | 63 | 76.8% |  |
|  | 1 | 6 | 21.4% | 8 | 14.8% | 14 | 17.1% |  |
|  | 2 | 1 | 3.6% | 4 | 7.4% | 5 | 6.1% |  |
| **Post- treatment** | **Total** | **22** | **100.0%** | **53** | **100.0%** | **75** | **100.0%** | 0.30 |
|  | 0 | 11 | 50.0% | 30 | 56.6% | 41 | 54.7% |  |
|  | 1 | 6 | 27.3% | 18 | 34.0% | 24 | 32.0% |  |
|  | 2 | 5 | 22.7% | 5 | 9.4% | 10 | 13.3% |  |
| **3 month post-treatment** | **Total** | **26** | **100.0%** | **53** | **100.0%** | **79** | **100.0%** | 0.74 |
|  | 0 | 15 | 57.7% | 35 | 66.0% | 50 | 63.3% |  |
|  | 1 | 9 | 34.6% | 14 | 26.4% | 23 | 29.1% |  |
|  | 2 | 2 | 7.7% | 4 | 7.5% | 6 | 7.6% |  |

**Supplementary Table 3 -** **Worst CTCAE grade treatment emergent adverse events**

A treatment-emergent adverse event is defined as an event not present prior to the initiation of trial treatment or an event already present that worsens at end of treatment or at 3 month follow-up.

|  | **Surgery** | | **Chemoablation** | | **Total** | |
| --- | --- | --- | --- | --- | --- | --- |
|  | **N** | **%** | **N** | **%** | **N** | **%** |
| **Total** | **28** | **100.0%** | **53** | **100.0%** | **81** | **100.0%** |
| **CTCAE grade** |  |  |  |  |  |  |
| 0 | 15 | 53.6% | 25 | 47.2% | 40 | 49.4% |
| 1 | 8 | 28.6% | 21 | 39.6% | 29 | 35.8% |
| 2 | 5 | 17.9% | 7 | 13.2% | 12 | 14.8% |
| Chi-square p-value: 0.59 | | | | | | |

**Supplementary Table 4 - Worst CTCAE grade treatment emergent adverse events by type of event**

|  | **CTCAE  grade** | **Surgery** | | **Chemoablation** | | **p-value*** |
| --- | --- | --- | --- | --- | --- | --- |
|  |  | **N** | **%** | **N** | **%** |  |
| **Total patients** |  | **28** | **100.0%** | **53** | **100.0%** |  |
| Anorexia | 1 | 1 | 3.6% | 0 | 0.0% | 0.35 |
| Bladder infection | 2 | 3 | 10.7% | 0 | 0.0% | 0.02 |
| Bladder spasm discomfort | 1 | 4 | 14.3% | 2 | 3.8% | 0.09 |
| Haematuria | 1 | 3 | 10.7% | 3 | 5.7% | 0.34 |
|  | 2 | 2 | 7.1% | 1 | 1.9% |  |
| Malaise | 1 | 4 | 14.3% | 2 | 3.8% | 0.18 |
| Nausea | 1 | 1 | 3.6% | 3 | 5.7% | 0.65 |
|  | 2 | 0 | 0.0% | 1 | 1.9% |  |
| Platelet count decreased | 1 | 0 | 0.0% | 1 | 1.9% | 0.81 |
| Rash | 1 | 0 | 0.0% | 3 | 5.7% | 0.29 |
|  | 2 | 0 | 0.0% | 1 | 1.9% |  |
| Urinary frequency | 1 | 8 | 28.6% | 9 | 17.0% | 0.27 |
|  | 2 | 1 | 3.6% | 1 | 1.9% |  |
| Urinary incontinence | 1 | 1 | 3.6% | 0 | 0.0% | 0.12 |
|  | 2 | 1 | 3.6% | 0 | 0.0% |  |
| Urinary obstruction | 1 | 1 | 3.6% | 2 | 3.8% | >0.99 |
| Urinary retention | 1 | 2 | 7.1% | 3 | 5.7% | >0.99 |
| Urinary tract pain | 1 | 6 | 21.4% | 4 | 7.5% | 0.10 |
|  | 2 | 1 | 3.6% | 1 | 1.9% |  |
| Urinary urgency | 1 | 6 | 21.4% | 6 | 11.3% | 0.13 |
|  | 2 | 2 | 7.1% | 1 | 1.9% |  |
| *Other conditions reported* |  |  |  |  |  |  |
| Diarrhoea | 1 | 1 | 3.6% | 1 | 1.9% | na |
| Abdominal pain | 1 | 1 | 3.6% | 0 | 0.0% |  |
| Back pain | 1 | 1 | 3.6% | 0 | 0.0% |  |
| Candida infection | 2 | 0 | 0.0% | 1 | 1.9% |  |
| Cough | 1 | 1 | 3.6% | 0 | 0.0% |  |
| Epistaxis | 2 | 0 | 0.0% | 1 | 1.9% |  |
| Fatigue | 1 | 0 | 0.0% | 1 | 1.9% |  |
| Feeling of body temperature change | 1 | 0 | 0.0% | 1 | 1.9% |  |
| Gouty arthritis | 2 | 0 | 0.0% | 1 | 1.9% |  |
| Headache | 1 | 0 | 0.0% | 1 | 1.9% |  |
| Labyrinthitis | 1 | 0 | 0.0% | 1 | 1.9% |  |
| Nocturia | 1 | 0 | 0.0% | 1 | 1.9% |  |
| Polymyalgia rheumatica | 1 | 0 | 0.0% | 1 | 1.9% |  |
| Pruritus | 1 | 0 | 0.0% | 1 | 1.9% |  |

*Fisher exact test comparing Gr1+ vs Gr 0 or missing.

**Supplementary Figure 1 – HRQOL** **Change from baseline in QLQ-C30 Physical function**

High score represents high quality of life
Positive change from baseline (computed as timepoint – baseline scores) represents improvement.

**Supplementary Figure 2 – HRQOL** **Change from baseline in QLQ-NMIBC24 Urinary symptoms**

High score represents worse urinary symptoms. Positive change (computed as difference baseline – timepoint scores) represents improvement in symptoms.
